# The Synergistic Effects of Gold Particles and Dexamethasone on the Electrochemical and Biological Performance of PEDOT Neural Interfaces

**DOI:** 10.3390/polym11010067

**Published:** 2019-01-05

**Authors:** Katarzyna Krukiewicz, Magdalena Chudy, Stephen Gregg, Manus J. P. Biggs

**Affiliations:** 1 Centre for Research in Medical Devices, National University of Ireland Galway, Newcastle Road, H91 W2TY Galway, Ireland; s.gregg060@gmail.com (S.G.); manus.biggs@nuigalway.ie (M.J.P.B.); 2 Department of Physical Chemistry and Technology of Polymers, Silesian University of Technology, M. Strzody 9, 44-100 Gliwice, Poland; magdalenachu93@gmail.com

**Keywords:** neural interfaces, gold particles, dexamethasone, PEDOT

## Abstract

Although neural devices have shown efficacy in the treatment of neurodegenerative diseases, their functionality is limited by the inflammatory state and glial scar formation associated with chronic implantation. The aim of this study was to investigate neural electrode performance following functionalization with an anti-inflammatory coating derived from a conducting polymer poly(3,4-ethylenedioxythiophene) (PEDOT) matrix doped with dexamethasone (Dex) and decorated with Au particles. Pristine PEDOT, PEDOT-Dex and their gold-decorated analogues (PEDOT/Au and PEDOT-Dex/Au) were formulated by electrochemical deposition and characterized with respect to electrode electrochemical properties, surface morphology and biocompatibility towards primary neural cells. Through a process of gold deposition, it was possible to eliminate the initial burst release observed in PEDOT-Dex and maintain a stable, stepwise increase in Dex elution over 7 days. The released amounts of Dex exceeded the concentrations considered as therapeutic for both PEDOT-Dex and PEDOT-Dex/Au. The results clearly indicated that the presence of either Dex or Au particles facilitated the outgrowth of neurites. Finally, it was shown that the application of composite materials, such as PEDOT-Dex/Au, is an efficient way to improve the efficacy of neural interfaces in vitro.

## 1. Introduction

Neuromodulatory devices have shown great promise for the treatment of neurodegenerative disorders, such as Parkinson disease, Alzheimer disease and Tourette’s syndrome [1]. By applying electrical stimulation to specific locations of the brain, neural interface devices have the ability to restore lost neural function [2]. The long-term implantation of a neuroelectrode, however, generates an inflammatory reaction, leading to peri-electrode encapsulation and a reduction in device performance [3,4]. Experimental approaches to reducing the foreign body response have focused on anti-inflammatory neuroelectrode coatings, which offer the potential to enhance electrode performance while providing a localized delivery of anti-inflammatory or neurotropic drugs [5].

Conducting polymers currently represent one of the most studied classes of neural interface materials, demonstrating advantageous electrical properties, high biocompatibility and drug delivery potential [6]. Due to their ion exchange properties, conducting polymers are able to immobilize ionic species and release them either through spontaneous dedoping or as a function of an applied electrical potential [6,7]. Dexamethasone (Dex) is a potent anti-inflammatory corticosteroid commonly used in the field of neural devices [8], mainly because of its ability to reduce tissue reactions following electrode implantation [9]. When administrated systemically, Dex was found to cause serious side effects, including myopathy and diabetes [10]. In order to minimize its adverse action, the local delivery of Dex has been postulated in numerous studies, and notably in conjunction with conducting polymers as Dex carriers [11,12,13,14,15]. Although conducting polymers have been confirmed as efficient carriers of multiple drugs in vitro, the incorporation of Dex has been observed to decrease their electrochemical performance in terms of a charge storage capacity [16]. A second limitation of drug-loaded conducting polymer systems is an associated burst release profile, which has been found to narrow the therapeutic window of the material in vivo. In the work of Stevenson et al. [12], for instance, even thought the substantial amount of Dex was loaded into a polyterthiophene conducting matrix, the therapeutic window was obtained at approximately 1 day in vitro, after which the concentration of released Dex reached a plateau. It follows that ongoing approaches to enhance the chronic performance of drug-loaded conducting matrices are focused on combinational physicochemical strategies [14,17,18]. In this paper, we postulate that a dual doping/composite approach, employing gold particles deposited onto the surface of a drug-loaded conducting polymer, will result in enhanced electrochemical performance and drug delivery potential. In particular, neural interfaces presenting gold particles have been observed to induce advantageous effects on the outgrowth, alignment and orientation of neurons in vitro [19,20] and the functionalization of an electrode surface with gold has become a promising approach to enhancing electrode cytocompatability. In this study, we hypothesized that by depositing Au particles on the surface of a Dex-loaded polymer matrix synergistic interactions would be observed, enhancing the electrochemical and biological performance of a resulting neural interface. Here, the efficacy of a Dex-loaded poly(3,4-ethylenedioxythiophene) (PEDOT) system functionalized through subsequent electrodeposition of submicron gold particles was evaluated in vitro. The as-formed matrices, i.e., pristine PEDOT, Dex-loaded polymer (PEDOT-Dex) and their gold-decorated analogues (PEDOT/Au and PEDOT-Dex/Au) were characterized in terms of their electrochemical properties, surface morphology and biocompatibility. The spontaneous elution of Dex was monitored over a period of 7 days, and the release kinetics was analyzed with the use of an Avrami’s mathematical model. The results of biological experiments were correlated with the chemistry of the surface as well as Dex elution profiles, providing the extensive characterization of a Dex-doped PEDOT system, gold-decorated PEDOT system, and the dual system exhibiting the synergistic effects of these two functionalities.

## 2. Materials and Methods

### 2.1. Fabrication of Poly(3,4-Ethylenedioxythiophene) (PEDOT)-Based Coatings

PEDOT-based coatings were fabricated through the process of electrochemical polymerization in a three electrode setup comprising Pt-coated Thermanox coverslip (Electron Microscopy Sciences, Hatfield, PA, USA) as a working electrode, Ag/AgCl (3 M KCl) (EDAQ, Sydney, Australia) as a reference electrode and a Pt-coated titanium rod (EDAQ, Sydney, Australia) as an auxiliary electrode, with the use of a PARSTAT 2273 potentiostat (Ametek, Berwyn, PA, USA). A cyclic voltammetric (CV) technique was used to polymerize 15 mM EDOT (Sigma Aldrich, St Louis, MO, USA) in the presence of 1× phosphate buffer solution (PBS, Sigma Aldrich, St Louis, MO, USA) and 10 mM Dexamethasone 21-phosphate disodium salt (Dex, Sigma Aldrich, St Louis, MO, USA). CV curves were collected at 100 mV/s for 20 CV cycles within the potential range from −0.8 to 1.5 V (vs. Ag/AgCl). A CV technique was also used to form a layer of gold particles on PEDOT-based coatings. CV was performed in a solution of 2 mM gold(III) chloride trihydrate (Sigma Aldrich, St Louis, MO, USA) in 1× PBS at 100 mV/s for 5 CV cycles within the potential ranging from 0.1 to −0.85 V (vs. Ag/AgCl).

### 2.2. Electrochemical Characterization

Electrochemical characterization of PEDOT-based coatings was performed with the use of a PARSTAT 2273 potentiostat in a three-electrode set-up, comprising a bare Pt foil or PEDOT-coated Pt-covered Thermanox coverslip as a working electrode, Ag/AgCl (3 M KCl) as a reference electrode and a Pt-coated titanium rod as an auxiliary electrode. Electrochemical impedance spectra (EIS) were collected in a 1× PBS solution within a frequency range from 100 MHz to 10 kHz, with AC amplitude of 40 mV (vs. Ag/AgCl) and DC potential equal to 0 V (vs. Ag/AgCl). The results were presented on Bode plots and compared to those of a bare Pt electrode. CV curves collected after polymerization were used to determine charge storage capacity (CSC), calculated according to the formula [7]:

(1) $$CSC=\int_{t_{1}}^{t_{2}}I\left(t\right)dt$$

where *t*_1_ is the beginning of a CV curve, *t*_2_ is the end of a CV cycle, and *I* is the current.

### 2.3. Chemical and Morphological Characterization

The chemical characterization of matrices was performed with the use of infrared spectroscopy. FTIR spectra were recorded using Varian 660-IR FT-IR Spectrometer (Agilent, San Jose, CA, USA) in the range between 4000 and 600 cm^−1^ for 16 scans. Morphological characterization was performed with the use of a Hitachi S-4700 scanning electron microscope, SEM (Hitachi, Tokyo, Japan) operating at 15 kV.

### 2.4. In Vitro Drug Release

The release experiments were conducted in 1× PBS. Pt-coated Thermanox coverslips coated with PEDOT, PEDOT/Au, PEDOT-Dex and PEDOT-Dex/Au were immersed in 1 mL of 1× PBS and left under gentle shaking (80 rpm, 37 °C) for 7 days. 0.1 mL of the supernatant was taken at a specific time points (1 h, 2 h, 4 h, 8 h, 12 h, 24 h, 2 days, 3 days, 4 days, 5 days, 6 days and 7 days) and every time 0.1 mL of a fresh PBS solution was added to keep the constant elution volume of 1 mL. This procedure prevented the saturation of the supernatant with Dex. The concentration of released Dex was determined through ultraviolet-visible (UV–Vis) spectrophotometry with the use of a Thermo Scientific Evolution 60S Spectrophtometer (Thermo Fisher Scientific, Waltham, MA, USA) and based on the absorbance at the peak characteristic for Dex (242 nm) [13]. UV–Vis spectra collected for PEDOT-Dex and PEDOT-Dex/Au were normalized by subtracting the absorbances collected for PEDOT. The measurements were performed for three technical replicas and the results were expressed as a mean ± standard deviation. The release kinetics was analyzed with the use of an Avrami’s equation:

(2) $$X=1-\exp\left(-kt^{n}\right)$$

and its linear form:

(3) ln(−ln(1−X))=lnk+nlnt

where: *X* is the fraction of drug released at time *t*, *n* is the Avrami parameter and *k* is the release rate constant [21].

### 2.5. Biological Characterization

The cytocompatibility of PEDOT, PEDOT/Au, PEDOT-Dex and PEDOT-Dex/Au was determined with respect to primary cultures of a mixed neural population obtained from the mesencephalon of embryonic Sprague–Dawley, and cultured for 7 days, as described previously [22,23]. All experiments were performed in accordance with the EU guidelines (2010/63/UE) and were approved by Health Products Regulatory Authority (AE19125/I179) and the local authority veterinary service. Every effort was made to minimize animal suffering and to reduce the number of animals used. The indirect double-immunofluorescent labelling was applied to visualize neuron and astrocyte cell populations and an Olympus Fluoview 1000 Confocal Microscope (Olympus, Tokyo, Japan) was used to obtain fluorescent images [23,24]. Cell density was analyzed by counting the number of nuclei corresponding to neurons and astrocytes in an area of 211.97 μm × 211.97 μm in at least 20 random images taken from test and control groups [24]. The quantification of neurite length was through stereological methods as reported previously [25], and the average neurite length was calculated according to the formula [26]:

(4) $$L=nT\frac{\pi }{2}$$

where *L* is neurite length (μm), *n* is the number of times neurites intersect with grid lines, *T* is distance between grid lines (μm).

The biological experiments were conducted to include three biological replicates for all the experimental groups. The results were expressed as the mean of the values ± standard error of the mean. A *t*-test was performed to determine the statistical significance (*p* < 0.05).

## 3. Results

### 3.1. Electrochemical Characterization

**Figure 1:**
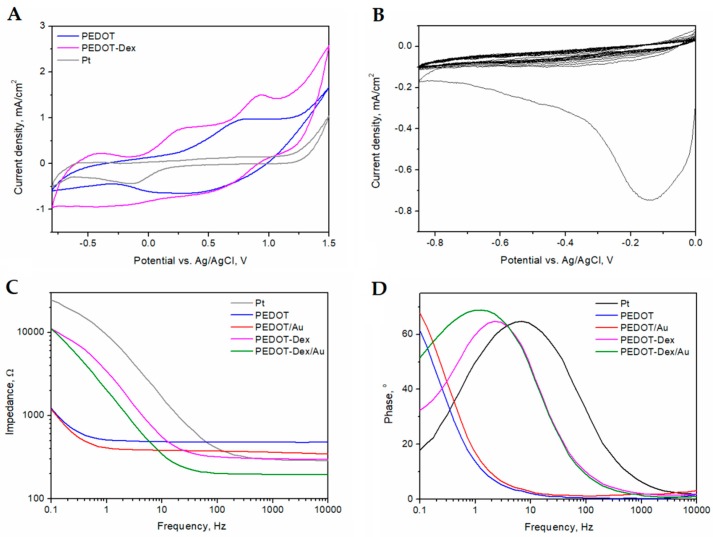
Electrochemical properties of poly(3,4-ethylenedioxythiphene) (PEDOT)-functionalized Pt electrodes. (**A**) Cyclic voltammetric (CV) curves of the final cycles of the electropolymerization process of EDOT (15 mM) formed in 1× phosphate buffered saline (PBS) or in 1× PBS in the presence of 10 mM Dex, together with a CV of a bare Pt electrode. (**B**) CV curves of the process of gold deposition from 2 mM HAuCl_4_ aqueous solution in 1× PBS. (**C**) Bode plot expression of impedance modulus and (**D**) Bode plot expression of the phase angle of PEDOT, PEDOT/Au, PEDOT-Dex, PEDOT-Dex/Au and a bare Pt electrode, collected in 1× PBS.

Figure 1A shows the final cycles of the electropolymerization process of PEDOT formed in PBS (PEDOT) and in the presence of PBS and Dex (PEDOT-Dex), as well as a CV curve of a Pt foil electrode. A well-defined oxidation peak was observed for PEDOT at 0.7 V (vs. Ag/AgCl) and reduction occurred within the range of 0 to 0.4 V (vs. Ag/AgCl), which has been previously observed for this polymer [27]. An oxidation peak at 0.9 V (vs. Ag/AgCl) was assigned to the oxidation of Dex, being facilitated by the presence of the conducting polymer matrix. CSC was found to achieve the values of 36 ± 8 mC/cm^2^ for PEDOT-Dex, 26 ± 7 mC/cm^2^ for PEDOT and 8 ± 1 mC/cm^2^ for a bare Pt.

It was observed that the incorporation of Dex had an effect on the electrochemical impedance of PEDOT coated electrodes (Figure 1C). PEDOT-Dex exhibited a lower impedance profile than that of pristine-coated PEDOT electrodes in the high frequency range (>20 Hz). Conversely, the impedance of PEDOT-Dex coated electrodes was observed to increase in the low frequency region, albeit it was still lower than the impedance of a bare Pt electrode. The changes in the charge transfer behavior of PEDOT-Dex coated electrodes when compared with PEDOT coated electrodes were also observed in the phase angle profile (Figure 1D) and the presence of a distinct peak for PEDOT-Dex at the frequency of 2 Hz.

The process of gold electrodeposition was performed according to [28], and the corresponding CV curves are presented in Figure 1B. The majority of gold was reduced in the first CV cycle, with an observed maximum reduction peak at −0.18 V (vs. Ag/AgCl), as reported in [28]. The lack of a distinct reduction peak in the next CV cycles was caused by the fact that the electrode reaction was limited by the diffusion of AuCl_4_^−^. The reduction of AuCl_4_^−^ onto the surface of PEDOT matrices resulted in the formation of gold particles, that are further characterized in Section 3.2. The presence of surface gold particles was shown to have the advantageous effects on the electrochemical characteristics of all experimental PEDOT coatings, especially with respect to electrode impedance (Figure 1C,D). With respect to PEDOT and PEDOT-Dex functionalized electrodes, the deposition of gold particles resulted in a decrease in the impedance profile over the whole range of frequencies investigated.

### 3.2. Surface Characterization

**Figure 2:**
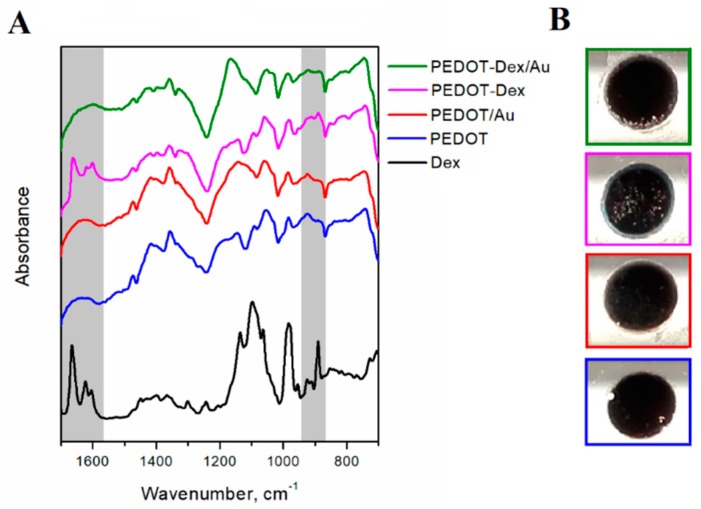
Surface characteristics of PEDOT functionalized Pt electrodes. (**A**) Fourier transform-infrared (FTIR) spectra and (**B**) macroscopic images of Dex, pristine PEDOT, PEDOT/Au, PEDOT-Dex and PEDOT-Dex/Au; grey regions in FTIR spectra indicate signals characteristic for Dex that can be found in PEDOT-Dex and PEDOT-Dex/Au.

In order to assess the Dex presence on the surface of PEDOT-Dex and PEDOT-Dex/Au functionalized electrodes, Fourier transform-infrared (FTIR) spectra were collected and analyzed (Figure 2A). All PEDOT-based coatings exhibited a set of signals typical for PEDOT, i.e., stretching vibrations of ethylenedioxy group (C–O–R–O–C) at 924, 1051 and 1185 cm^−1^ [29], C–C and C=C stretching in thienylene moiety (1472 and 1356 cm^−1^) [29,30], as well as the absorption band assigned to C–S vibrations (922 cm^−1^) and C–S–C deformation (711 cm^−1^) [30]. The most characteristic absorption bands of Dex in the FTIR spectrum are assigned to –C=O and double bond framework conjugated to –C=O bonds (1665 and 1620 cm^−1^) [31,32], as well as axial deformation of C–F group (890 cm^−1^) [33]. These peaks were found in the spectrum of PEDOT-Dex, as indicated by the grey background in Figure 2A, confirming that a significant amount of Dex was present on the polymer surface. Conversely, the FTIR spectrum of PEDOT-Dex/Au contained only a weak absorption band associated with C–F deformation, indicating that the amount of surface-immobilized Dex was significantly lower than was that associated with PEDOT-Dex substrate. Macroscopic images of PEDOT-based matrices (Figure 2B) indicate a uniform, continuous coating in the case of PEDOT, PEDOT/Au and PEDOT-Dex/Au, yet the presence of several cracks and holes were noted on the surface of the PEDOT-Dex substrate.

**Figure 3:**
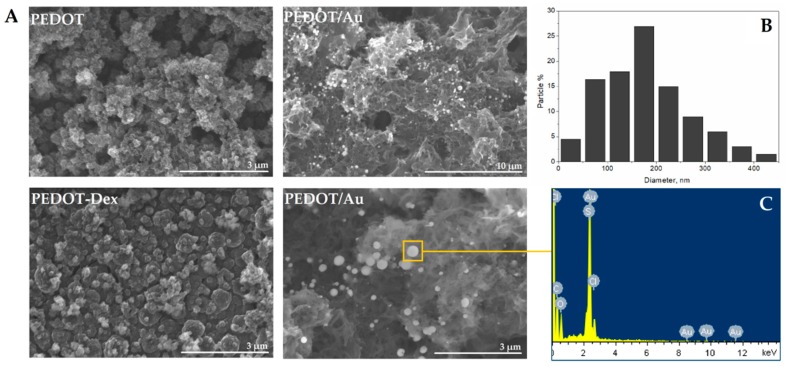
Surface morphology of Dex-functionalized PEDOT and PEDOT composite matrices. (**A**) Scanning electron microscope (SEM) images of PEDOT, PEDOT-Dex and PEDOT/Au matrices. (**B**) Size distribution of gold particles and (**C**) energy-dispersive X-ray (EDS) spectrum of a region of interest depicted in the SEM image.

SEM imaging (Figure 3A) revealed that PEDOT derived coatings possessed a porous and rough surface structure as previously reported [34]. Conducting electropolymerization in the presence of Dex induced a modest change in morphology and the formation of larger grains were observed [35,36]. When the polymer matrix was subjected to reduction in AuCl_4_^−^ solution, gold was deposited onto the surface, creating particles with a diameter from 30–440 nm (Figure 3B), with an average diameter of 170 nm. The chemical composition of the particles was revealed through energy-dispersive X-ray (EDS) spectra (Figure 3C), indicating the presence of gold and chloride, as well as carbon, oxygen and sulfur associated with PEDOT.

### 3.3. In Vitro Drug Release

Due to their ion-exchange properties, conducting polymers are able to immobilize charged molecules and release them in a controlled manner [6]. Negatively charged drugs, such as Dex, are typically immobilized during oxidative polymerization of EDOT, and consecutively released during the spontaneous dedoping of the polymer [6]. The kinetics of the release is the function of the physicochemical parameters of the polymer coating, such as its porosity, morphology and ion exchange capacity, as well as the properties of the drug itself, including ion size and mobility, and can be facilitated by an external stimuli, e.g., an electrical potential [34].

**Figure 4:**
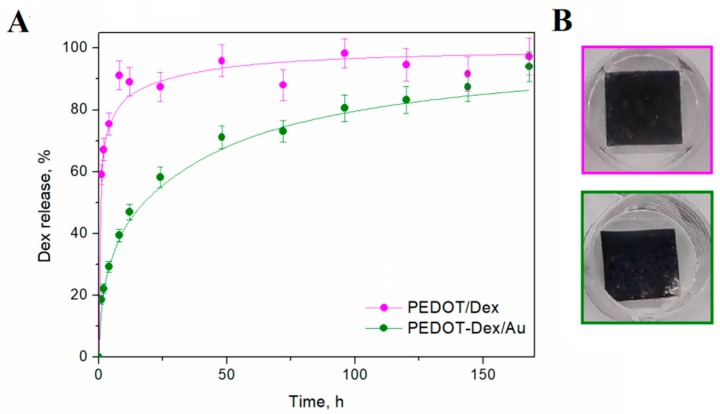
Dex release from Dex-doped PEDOT and PEDOT composite substrates. (**A**) Drug elution profiles of spontaneous Dex release from PEDOT-Dex and PEDOT-Dex/Au functionalized electrodes. Dots are the experimental values and lines represent the corresponding fitted curves calculated by means of Avrami’s equation. (**B**) Macroscopic images of PEDOT-Dex and PEDOT-Dex/Au functionalized electrodes after performing the elution experiment.

The release kinetics can be described through mathematical models, among which the Avrami’s model is frequently used for conducting polymer-based drug delivery systems [21,37,38,39,40]. Here, Avrami’s model was found to provide a reliable simulation of the release parameters for PEDOT-Dex and PEDOT-Dex/Au systems. The fitted release curves were plotted together with the experimental curves in Figure 4A and the release kinetic parameters were presented in Table 1. The highest release rate constant (0.95 1/h) was noted for the PEDOT-Dex system, but this system also exhibited an initial burst release. Consequently, it was observed that PEDOT-Dex functionalized electrodes released the total Dex payload after approximately 2 days, and extending the elution time did not result in any change in the cumulative concentration of released drug. Conversely, PEDOT-Dex/Au functionalized electrodes exhibited a more controlled release profile with a lower release rate constant (0.18 1/h) and a stepwise increase in Dex concentration over a 7-day period. The total amounts of released drug after 7 days were 17 and 13 μM/cm^2^ for PEDOT-Dex and PEDOT-Dex/Au, respectively. Macroscopic images of PEDOT-Dex and PEDOT-Dex/Au substrates (Figure 4B) indicated that the release process did not result in the formation of significant surface defects.

### 3.4. Biological Characterization

**Figure 5:**
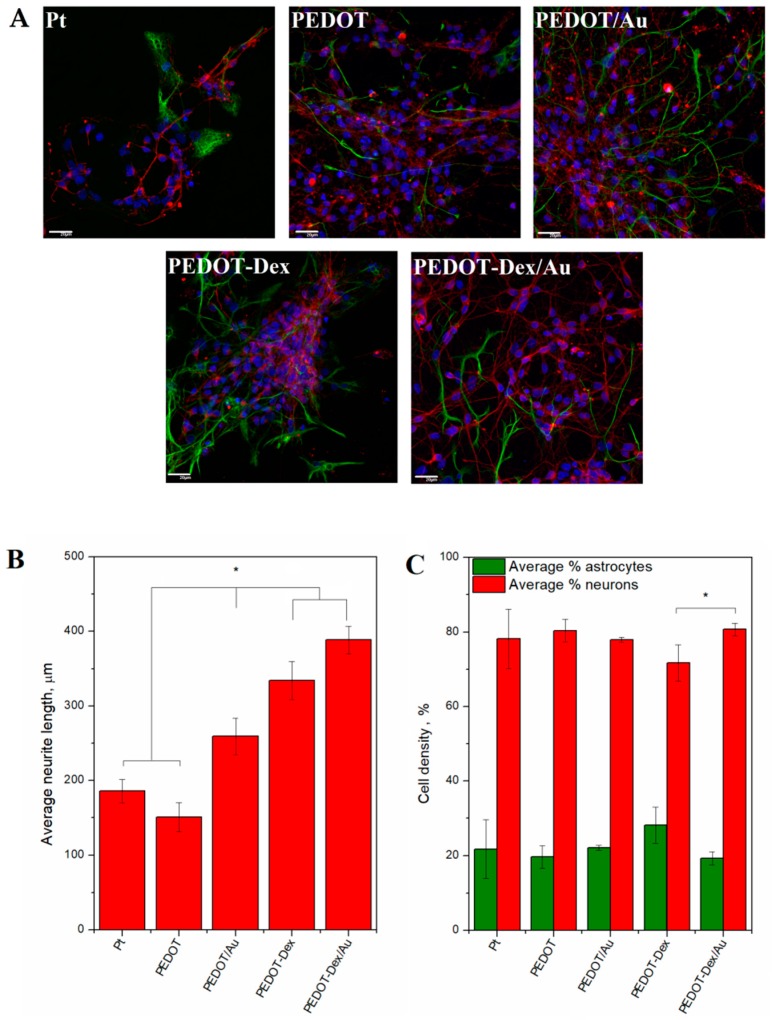
Biological properties of PEDOT functional coatings and bare Pt substrates. (**A**) Fluorescent images of primary ventral mesencephalic (VM) mixed cell population cultured for seven days on magnetron sputtered Pt, PEDOT, PEDOT/Au, PEDOT-Dex and PEDOT-Dex/Au; neurons are visualized by anti β-tubulin III (red), astrocyte cells by anti-glial fibrillary acidic protein, GFAP stain (green) and nuclei by 4′,6-diamidino-2-phenylindole, DAPI (blue). (**B**) The average neurite length and (**C**) cell density analysis of astrocyte and neuron presence on each of the experimental and control group; ★ = *p* < 0.05, N = 3.

The cytocompatibility of PEDOT-based coatings was assessed through culturing a primary ventral mesencephalic (VM) mixed cell population on the modified surfaces of Pt-coated Thermanox coverslip. VM cells were cultured for 7 days, and following that period they were fixed and immunostained with 4′,6-diamidino-2-phenylindole (DAPI) for cell nuclei, anti-β III tubulin for neurons and anti-glial fibrillary acidic protein (GFAP) for astrocytes (Figure 5A). The fluorescent images were then quantified to estimate the average neurite length (Figure 5B) and the neuron-to-astrocyte ratio (Figure 5C). The most extensive neuron outgrowth was noted for PEDOT-Dex/Au and PEDOT-Dex functionalized substrates, for which the average neuron lengths after 7 days in culture were 388 ± 19 and 334 ± 25 µm, respectively. The neural-to-astrocyte ratio, which is used to assess the cytocompatibility of a material in vitro with respect to neural systems [24], showed the prevalence of neurons on the surface of PEDOT-Dex/Au relative to PEDOT-Dex.

## 4. Discussion

The electropolymerization of EDOT in the presence of Dex was confirmed as an efficient way to form Dex-loaded conducting matrix, possessing high charge storage capacity (36 ± 8 mC/cm^2^) and low impedance (300 ± 48 Ω at 1 kHz), outperforming both pristine PEDOT as well as bare Pt electrodes. It is hypothesized that the observed increase in CSC in the presence of Dex, in contrast to a decrease in CSC as reported by Goding et al. [16], is as a result of the additional faradaic processes of Dex occurring under oxidative conditions.

According to [41], the potential of 1.05 V (vs. Ag/AgCl) was enough to trigger the electro-oxidation of Dex, in which the –OH group present at C11 was oxidized to form a ketone. The as-formed 11-ketodexamethasone was still able to act as a potent glucocorticoid receptor agonist, with its efficiency comparable to Dex [42]. The presence of drug was also shown to cause the shift of the oxidation peak of PEDOT to a lower potential (0.25 V vs. Ag/AgCl), as well as the expected increase in the capacitance of polymer-coated electrodes. The changes in the impedance profile and phase angle behavior when Dex was immobilized within PEDOT should be associated with the additional capacitance of the system that was caused by a diffusion of Dex [14,43]. Nevertheless, the fact that the impedance behavior of PEDOT-Dex resembled more Pt than PEDOT substrates can be attributed to the presence of cracks and holes on the surface of a drug-loaded polymer, exposing regions of the non-coated Pt electrode, and indicating that PEDOT-Dex is prone to cracking and delamination as observed previously [16]. The obtained FTIR spectra of PEDOT-Dex films confirmed that Dex molecules were present on its surface, leading to the formation of larger polymer grains relative to pristine PEDOT films, possibly resulting from the strong interactions between EDOT and Dex serving as a co-dopant [35,36]. The presence of Dex on the surface of the polymer resulted in a rapid and spontaneous drug elution, leading to an observed initial burst release in the elution profile of this matrix.

In order to improve the electrochemical properties and elution profile of PEDOT-based electrode coatings, gold particles with an average diameter of 170 nm were electrodeposited on top of PEDOT and PEDOT-Dex films. According to Elsabahy et al. [44], the observed diameter of the deposited gold particles was within a range which yields the highest potential for in vivo applications. The presence of gold resulted in a decrease in the impedance profile over the whole range of investigated frequencies. This was supposed to be the effect of the surface morphology, as observed in previous investigations on the electrodeposition of gold on the surface of gold electrodes [45]. Interestingly, the surface of Au-coated Dex-functionalized electrodes was evidently more homogeneous than PEDOT-Dex substrates with no traces of cracking or delamination. The resulting impedance and phase profiles showed that PEDOT-Dex/Au substrate combines the beneficial electrochemical properties of both drug-loaded and Au-functionalized surfaces, exhibiting low impedance at high frequencies (typical for highly conducting metals) with low impedance at low frequencies (typical for highly capacitive conducting polymers), confirming a synergistic interaction between dexamethasone-loaded conducting polymer matrix and a surface functionalization with gold particles.

Since the process of electrodeposition required the application of negative potentials, some of the easily accessible surface Dex molecules were removed from PEDOT-Dex matrix during gold deposition, which was observed with PEDOT-Dex/Au films as an absence of the characteristic Dex FTIR signal. This surface depletion of Dex in PEDOT-Dex/Au films resulted in the elimination of an initial burst release of Dex and decreased the rate of passive drug release, minimizing the risk of elevated local Dex concentrations which could be both pharmacologically dangerous and economically inefficient [46]. Moreover, the stability of PEDOT-Dex/Au coating was confirmed by retaining the surface uniformity after the release process in vitro. The release kinetics was found to fit well to the Avrami’s model of spontaneous release, exhibiting a more controlled release profile with lower release rate constant (0.18 1/h) than was the case with PEDOT-Dex coatings (0.95 1/h). The value of the Avrami parameter (n) close to 0.5 indicated that Dex elution from the PEDOT-Dex/Au matrix corresponded to a diffusive release state [37]. Basing on the established kinetic release model, it was calculated that the total amount of Dex immobilized in PEDOT/-Dex/Au substrates would be released after 30 days in vitro, indicating the potency of this system for the extended drug release. Conversely, the therapeutic window of a PEDOT-Dex system was estimated to persist for only 12 days in vitro, due to a rapid release of Dex in an initial burst phase. Critically, Dex concentrations of 0.2 μM have been shown to effectively reduce the inflammatory tissue reactions around a neural implant [9,47], indicating the released amounts of Dex exceeded the concentrations considered as therapeutic for both PEDOT-Dex and PEDOT-Dex/Au matrices (17 and 13 μM/cm^2^, respectively). Other studies on Dex-loaded conducting polymer systems showed the release values ranging from 6 μg/cm^2^ [48] to 16 μg/cm^2^ [11] in case of polypyrrole-based systems, and approx. 23 μM [13] for PEDOP (when Dex was used in a similar initial concentration). In the work of Stevenson et al. [12] the substantial amount of 80 μg/cm^2^ (approximately 50 μmol) was released from a polyterthiophene (PTTh) matrix, but the release experiment was performed for 1400 min (approx 1 day), after which the concentration reached plateau. Although the observed amounts of released Dex for PEDOT-Dex/Au functionalized electrodes were lower, the release curve was found to fit well to the diffusion model, confirming its controlled character. Moreover, PEDOT-Dex/Au did not exhibit an initial burst release, which was the limitation of a PTTh–Dex [12].

All experimental and control functional chemistries were found to influence the growth of neural cells and in vitro analysis clearly indicated that the presence of Dex and Au particles had a positive effect on neurite outgrowth, which was further enhanced with combination materials. Although PEDOT coating did not significantly effect neurite outgrowth relative to Pt-coated substrates, PEDOT/Au coatings were observed to outperform pristine PEDOT coatings by increasing the average neurite length by approx. 50%. This confirmed an advantageous effect of the presence of gold particles on cell outgrowth, as described previously [19,20]. The neurite length in neurons cultured on PEDOT-Dex and PEDOT-Dex/Au, however, was significantly increased relative to control Pt films, pristine PEDOT and PEDOT-Au films, with a prevalence of neurons on the surface of PEDOT-Dex/Au with respect to PEDOT-Dex. It is hypothesized that this pro-neural effect was a result of PEDOT-Dex matrix conditioning occurring during the deposition of Au. In this scenario, Au electrodeposition cleared loosely attached Dex from the PEDOT surface and negated an initial burst release of Dex, preventing its negative effect on the neuronal population [46]. Nevertheless, it has been shown that both functionalization strategies, drug-loading and Au-deposition resulted in an improvement in the biological performance of the PEDOT interface, with a synergistic effect observed through employing dual functionalisation. It can be hypothesized that electrochemical deposition of gold particles may represent an interesting option for further improvement to the performance of other drug-loaded conducting polymer systems, including polypyrrole [11] and polyterthiophene [12].

## 5. Conclusions

In summary, through the employment of Dex doping into a PEDOT-conducting polymer matrix and subsequent gold particle functionalization, a system exhibiting synergistic effects of these two functionalities was developed. The advantageous electrochemical characteristics accompanied a therapeutically relevant spontaneous drug release, which resulted in an enhancement of neural growth in vitro relative to other experimental and control systems explored in this study. Finally, it has been shown that the a dual doping/composite approach to neural interface design represents an efficient strategy to improve the efficacy of neural interfaces in vivo.

## Figures and Tables

**Table 1:** Release kinetic parameters calculated basing on Avrami’s equation for the spontaneous release of Dex from PEDOT-Dex and PEDOT-Dex/Au.

Polymer Matrix	*k*, 1/h	*n*	*R* ^2^
PEDOT-Dex	0.95	0.28	0.94
PEDOT-Dex/Au	0.18	0.49	0.99

## References

[1] Chen X.L., Xiong Y.Y., Xu G.L., Liu X.F. (2013). Deep Brain Stimulation. Interv. Neurol..

[2] Shih J.J., Krusienski D.J., Wolpaw J.R. (2012). Brain-computer interfaces in medicine. Mayo Clin. Proc..

[3] Vallejo-Giraldo C., Kelly A., Biggs M.J.P. (2014). Biofunctionalisation of electrically conducting polymers. Drug Discov. Today.

[4] McConnell G.C., Rees H.D., Levey A.I., Gutekunst C.A., Gross R.E., Bellamkonda R.V. (2009). Implanted neural electrodes cause chronic, local inflammation that is correlated with local neurodegeneration. J. Neural Eng..

[5] Zhong Y., McConnell G.C., Ross J.D., Deweerth S.P., Bellamkonda R.V. A novel dexamethasone-releasing, anti-inflammatory coating for neural implants. Proceedings of the 2nd International IEEE EMBS Conference on Neural Engineering.

[6] Svirskis D., Travas-Sejdic J., Rodgers A., Garg S. (2010). Electrochemically controlled drug delivery based on intrinsically conducting polymers. J. Control. Release.

[7] Krukiewicz K., Zak J.K. (2014). Conjugated polymers as robust carriers for controlled delivery of anti-inflammatory drugs. J. Mater. Sci..

[8] Carli S., Trapella C., Armirotti A., Fantinati A., Ottonello G., Scarpellini A., Prato M., Fadiga L., Ricci D. (2018). Biochemically Controlled Release of Dexamethasone Covalently Bound to PEDOT. Chem.-A Eur. J..

[9] Zhong Y., Bellamkonda R.V. (2007). Dexamethasone-coated neural probes elicit attenuated inflammatory response and neuronal loss compared to uncoated neural probes. Brain Res..

[10] Koehler P.J. (1995). Use of corticosteroids in neuro-oncology. Anticancer Drugs.

[11] Wadhwa R., Lagenaur C.F., Cui X.T. (2006). Electrochemically controlled release of dexamethasone from conducting polymer polypyrrole coated electrode. J. Control. Release.

[12] Stevenson G., Moulton S.E., Innis P.C., Wallace G.G. (2010). Polyterthiophene as an electrostimulated controlled drug release material of therapeutic levels of dexamethasone. Synth. Met..

[13] Krukiewicz K. Tailorable drug capacity of dexamethasone-loaded conducting polymer matrix. Proceedings of the IOP Conference Series: Materials Science and Engineering.

[14] Castagnola E., Carli S., Vomero M., Scarpellini A., Prato M., Goshi N., Fadiga L., Kassegne S., Ricci D. (2017). Multilayer poly(3,4-ethylenedioxythiophene)-dexamethasone and poly(3,4-ethylenedioxythiophene)-polystyrene sulfonate-carbon nanotubes coatings on glassy carbon microelectrode arrays for controlled drug release. Biointerphases.

[15] Boehler C., Kleber C., Martini N., Xie Y., Dryg I., Stieglitz T., Hofmann U.G., Asplund M. (2017). Actively controlled release of Dexamethasone from neural microelectrodes in a chronic in vivo study. Biomaterials.

[16] Goding J.A., Gilmour A.D., Martens P.J., Poole-Warren L.A., Green R.A. (2015). Small bioactive molecules as dual functional co-dopants for conducting polymers. J. Mater. Chem. B.

[17] Abidian M.R., Kim D.H., Martin D.C. (2006). Conducting-polymer nanotubes for controlled drug release. Adv. Mater..

[18] Massoumi B., Entezami A. (2002). Electrochemically controlled binding and release of dexamethasone from conducting polymer bilayer films. J. Bioact. Compat. Polym..

[19] Baranes K., Shevach M., Shefi O., Dvir T. (2016). Gold Nanoparticle-Decorated Scaffolds Promote Neuronal Differentiation and Maturation. Nano Lett..

[20] Demir U.S., Shahbazi R., Calamak S., Ozturk S., Gultekinoglu M., Ulubayram K. (2018). Gold nano-decorated aligned polyurethane nanofibers for enhancement of neurite outgrowth and elongation. J. Biomed. Mater. Res. Part A.

[21] Shamaeli E., Alizadeh N. (2013). Kinetic studies of electrochemically controlled release of salicylate from nanostructure conducting molecularly imprinted polymer. Electrochim. Acta.

[22] Vallejo-Giraldo C., Pampaloni N.P., Pallipurath A.R., Mokarian-Tabari P., O’Connell J., Holmes J.D., Trotier A., Krukiewicz K., Orpella-Aceret G., Pugliese E. (2017). Preparation of Cytocompatible ITO Neuroelectrodes with Enhanced Electrochemical Characteristics Using a Facile Anodic Oxidation Process. Adv. Funct. Mater..

[23] Krukiewicz K., Chudy M., Vallejo-Giraldo C., Skorupa M., Więcławska D., Turczyn R., Biggs M. (2018). Fractal form PEDOT/Au assemblies as thin-film neural interface materials. Biomed. Mater..

[24] Vallejo-Giraldo C., Pugliese E., Larrañaga A., Fernandez-Yague M.A., Britton J.J., Trotier A., Tadayyon G., Kelly A., Rago I., Sarasua J.R., Dowd E., Quinlan L.R., Pandit A., Biggs M.J.P. (2016). Polyhydroxyalkanoate/carbon nanotube nanocomposites: Flexible electrically conducting elastomers for neural applications. Nanomedicine.

[25] O’Keeffe G.W., Dockery P., Sullivan A.M. (2004). Effects of growth/differentiation factor 5 on the survival and morphology of embryonic rat midbrain dopaminergic neurones in vitro. J. Neurocytol..

[26] Kavanagh E.T., Loughlin J.P., Herbert K.R., Dockery P., Samali A., Doyle K.M., Gorman A.M. (2006). Functionality of NGF-protected PC12 cells following exposure to 6-hydroxydopamine. Biochem. Biophys. Res. Commun..

[27] Estrany F., Oliver R., Armelin E., Iribaren J.I., Liesa F., Aleman C. (2007). Electroactive Properties and Electrochemical Stability of Poly(3,4-ethylenedioxythiophene) and Poly(N-methylpyrrole) Multi-layered Films Generated by Anodic Oxidation. Port. Electrochim. Acta.

[28] Hariri M.B., Dolati A., Moakhar R.S. (2013). The Potentiostatic Electrodeposition of Gold Nanowire/Nanotube in HAuCl4 Solutions Based on the Model of Recessed Cylindrical Ultramicroelectrode Array. J. Electrochem. Soc..

[29] Cui Z., Coletta C., Rebois R., Baiz S., Gervais M., Goubard F., Aubert P.H., Dazzi A., Remita S. (2016). Radiation-induced reduction-polymerization route for the synthesis of PEDOT conducting polymers. Radiat. Phys. Chem..

[30] Coletta C., Cui Z., Dazzi A., Guigner J.M., Néron S., Marignier J.L., Remita S. (2016). A pulsed electron beam synthesis of PEDOT conducting polymers by using sulfate radicals as oxidizing species. Radiat. Phys. Chem..

[31] Rodrigues L.B., Leite H.F., Yoshida M.I., Saliba J.B., Junior A.S.C., Faraco A.A.G. (2009). In vitro release and characterization of chitosan films as dexamethasone carrier. Int. J. Pharm..

[32] Wang C., Hou H., Nan K., Sailor M.J., Freeman W.R., Cheng L. (2014). Intravitreal controlled release of dexamethasone from engineered microparticles of porous silicon dioxide. Exp. Eye Res..

[33] Da Silva G.R., Da Silva-Cunha A., Behar-Cohen F., Ayres E., Oréfice R.L. (2011). Biodegradable polyurethane nanocomposites containing dexamethasone for ocular route. Mater. Sci. Eng. C.

[34] Krukiewicz K., Kruk A., Turczyn R. (2018). Evaluation of drug loading capacity and release characteristics of PEDOT/naproxen system: Effect of doping ions. Electrochim. Actarochim. Acta..

[35] Gao Y., Zhao L., Li C., Shi G. (2006). Electrosynthesis of poly(3,4-ethylenedioxythiophene) microcups in the aqueous solution of LiClO_4_ and tri(ethylene glycol). Polymer.

[36] Mumtaz M., Ibarboure E., Labrugère C., Cloutet E., Cramail H. (2008). Synthesis of PEDOT Nano-objects Using Poly(vinyl alcohol)-Based Reactive Stabilizers in Aqueous Dispersion. Macromolecules.

[37] Alizadeh N., Shamaeli E. (2014). Electrochemically controlled release of anticancer drug methotrexate using nanostructured polypyrrole modified with cetylpyridinium: Release kinetics investigation. Electrochim. Acta.

[38] Howe E.J., Okesola B.O., Smith D.K. (2015). Self-assembled sorbitol-derived supramolecular hydrogels for the controlled encapsulation and release of active pharmaceutical ingredients. Chem. Commun..

[39] Shamaeli E., Alizadeh N. (2014). Nanostructured biocompatible thermal/electrical stimuli-responsive biopolymer-doped polypyrrole for controlled release of chlorpromazine: Kinetics studies. Int. J. Pharm..

[40] Alizadeh N., Shamaeli E., Fazili M. (2017). Online Spectroscopic Monitoring of Drug Release Kinetics from Nanostructured Dual-Stimuli-Responsive Conducting Polymer. Pharm. Res..

[41] Demir E., Inam O., Inam R., Aboul-Enein H.Y. (2018). Voltammetric Determination of Ophthalmic Drug Dexamethasone Using Poly-glycine Multi Walled Carbon Nanotubes Modified Paste Electrode. Curr. Anal. Chem..

[42] Rebuffat A.G., Tam S., Nawrocki A.R., Baker M.E., Frey B.M., Frey F.J., Odermatt A. (2004). The 11-ketosteroid 11-ketodexamethasone is a glucocorticoid receptor agonist. Mol. Cell. Endocrinol..

[43] Abidian M.R., Martin D.C. (2008). Experimental and theoretical characterization of implantable neural microelectrodes modified with conducting polymer nanotubes. Biomaterials.

[44] Elsabahy M., Wooley K.L. (2012). Design of polymeric nanoparticles for biomedical delivery applications. Chem. Soc. Rev..

[45] Cui X., Martin D.C. (2003). Fuzzy gold electrodes for lowering impedance and improving adhesion with electrodeposited conducting polymer films. Sens. Actuators A Phys..

[46] Huang X., Brazel C.S. (2001). On the importance and mechanisms of burst release in matrix-controlled drug delivery systems. J. Control. Release.

[47] Shain W., Spataro L., Dilgen J., Haverstick K., Retterer S., Isaacson M., Saltzman M., Turner J.N. (2003). Controlling cellular reactive responses around neural prosthetic devices using peripheral and local intervention strategies. IEEE Trans. Neural Syst. Rehabil. Eng..

[48] Moulton S.E., Imisides M.D., Shepherd R.L., Wallace G.G. (2008). Galvanic coupling conducting polymers to biodegradable Mg initiates autonomously powered drug release. J. Mater. Chem..

